# Sentence-level multi-modal feature learning for depression recognition

**DOI:** 10.3389/fpsyt.2025.1439577

**Published:** 2025-03-21

**Authors:** Guanghua Zhang, Guangping Zhuo, Yang Yang, Guohua Xu, Shukui Ma, Hao Liu, Zhiyong Ren

**Affiliations:** ^1^ School of Big Data Intelligent Diagnosis & Treatment industry, Taiyuan University, Taiyuan, China; ^2^ Shanxi Key Laboratory of Intelligent Optimization Computing and Blockchain Technology, Taiyuan Normal University, Taiyuan, Shanxi, China; ^3^ Shanxi Eye Hospital, Taiyuan, Shanxi, China; ^4^ College of Computer Science and Technology, Taiyuan Normal University, Taiyuan, Shanxi, China; ^5^ Shanxi Tongchuang Technology Inc., Taiyuan, China; ^6^ Shanxi Province Mental Health Center, Taiyuan Psychiatric Hospital, Taiyuan, China

**Keywords:** depression recognition, multi-modal feature learning, sentence-level, cross-modal joint attention, binary depression classification

## Abstract

**Background:**

The global prevalence of depression has escalated, exacerbated by societal and economic pressures. Current diagnostic methodologies predominantly utilize single-modality data, which, despite the existence of certain multi-modal strategies, often fail to effectively harness the distinct contributions of each modality in depression detection.

**Methods:**

This study collected multi-modal features from 100 participants (67 depressed patients and 33 non-depressed individuals) to formulate a MMD2023 dataset, and introduces the Sentence-level Multi-modal Feature Learning (SMFL) approach, an automated system designed to enhance depression recognition. SMFL analyzes synchronized sentence-level segments of facial expressions, vocal features, and transcribed texts obtained from patient-doctor interactions. It incorporates Temporal Convolutional Networks (TCN) and Long Short-Term Memory (LSTM) networks to meticulously extract features from each modality, aligned with the structured temporal flow of dialogues. Additionally, the novel Cross-Modal Joint Attention (CMJAT) mechanism is developed to reconcile variances in feature representation across modalities, adeptly adjusting the influence of each modality and amplifying weaker signals to equate with more pronounced features.

**Results:**

Validated on our collected MMD2023 dataset and a public available DAIC-WOZ containing 192 patients dataset, the SMFL achieves accuracies of 91% and 89% respectively, demonstrating superior performance in binary depression classification. This advanced approach not only achieves a higher precision in identifying depression but also ensures a balanced and unified multi-modal feature representation.

**Conclusion:**

The SMFL methodology represents a significant advancement in the diagnostic processes of depression, promising a cost-effective, private, and accessible diagnostic tool that aligns with the PHQ-8 clinical standard. By broadening the accessibility of mental health resources, this methodology has the potential to revolutionize the landscape of psychiatric evaluation, augmenting the precision of depression identification and enhancing the overall mental health management infrastructure.

## 1 Introduction

In recent years, depression has emerged as a predominant global health concern, affecting approximately 280 million individuals worldwide, according to the World Health Organization (WHO) (1). The COVID-19 pandemic has exacerbated this issue, leading to a 27% increase in depression cases due to widespread insecurity about finances, physical health, and mental well-being (2). Depression not only affects individuals by engendering persistent sadness, lack of interest, and negative thoughts but also imposes significant economic and social costs on society by reducing workforce productivity and increasing the risk of suicide, with over 800,000 related deaths annually (3).

The conventional diagnosis of depression involves subjective assessments during patient interviews, considering factors like speech tone, facial expressions, and eye movements. Tools such as the PHQ-8 and HAMD are frequently used (4); however, they require extensive time and experienced clinical judgment, making the diagnosis process cumbersome and prone to bias. Consequently, there is a critical need for more efficient, objective methods to support early diagnosis and reduce reliance on subjective evaluations.

Addressing this need, recent advancements have been made in depression identification using deep learning on both single-modal (5–8) and multi-modal data (9–15). For instance, employing short-term speech segments (16) or integrating various acoustic features (17) through deep learning models has shown promise. Additionally, temporal convolutional networks have been used to analyze multi-modal data such as facial expressions, speech signals, and transcribed texts, enhancing diagnosis accuracy (18).

Despite these advancements, the integration of data from extended patient-doctor interactions remains challenging due to the dynamic and inconsistent nature of different modalities, particularly in capturing weakly expressed features. To overcome these limitations, this paper introduces a novel architecture, the Sentence-level Multi-modal Feature Learning (SMFL) approach. SMFL utilizes dialogue-based timestamps to align features from multiple modalities, employing Temporal Convolutional Networks (TCN) and Long Short-Term Memory (LSTM) modules to process segmented features and enhance inter-sentence relationships. Additionally, our Cross-Modal Joint Attention (CMJAT) mechanism, leverages strong features such as text feature to amplify weak ones (e.g., video feature), significantly improving diagnostic accuracy.

Furthermore, this paper discusses the construction of the MMD2023 dataset in collaboration with several hospitals to address the scarcity of Chinese depression databases, providing a robust foundation for validating our model. The effectiveness of SMFL has been demonstrated through comprehensive experimental analyses on both the DAIC-WOZ and MMD2023 datasets, showing promising results in the field of depression identification.

## 2 Materials and methods

### 2.1 Data collection

In this study, we primarily collected data from the MMD2023 dataset, which was carefully curated in collaboration with the Fifth People’s Hospital of Taiyuan City. This dataset consists of detailed multi-modal data from 100 participants, including 67 individuals diagnosed with depression and 33 non-depressed control group participants. The MMD2023 dataset captures intricate interactions through simulated in-depth patient-doctor dialogues.

Data samples were meticulously collected by professional psychiatrists through clinical interviews and other diagnostic methods. The patients were assessed in structured questionnaire scores and the clinical expertise of the psychiatrists. Upon obtaining written informed consent from the patients, the sessions were conducted in a private diagnostic room, utilizing a portable high-resolution camera (Sony HDR-CX680) to record the interactions. Audio was captured at a low-pass filter sampling rate of 75 kHz, while video was recorded at a frame rate of 50 fps. Textual data were subsequently transcribed from the audio recordings using specialized transcription tools. To ensure the accuracy of the textual information, researchers verified and confirmed the transcribed data rigorously.

The ethical review process for this study adhered strictly to the guidelines established for the collection of depression-related data in China and complied with the Declaration of Helsinki and relevant national regulations, ensuring that the data collection process upheld principles of beneficence, non-maleficence, and justice. The study was approved by the Institutional Review Board (IRB) of Taiyuan Psychiatric Hospital (Approval #: 202018), which rigorously evaluated the study protocol for compliance with ethical standards. Participants were first briefed on the study’s objectives, methods, and potential risks, followed by obtaining their informed consent. Confidentiality was maintained by anonymizing all personal identifiers and securely storing data.

To further validate the robustness and generalizability of our proposed method, we also utilized the publicly available DAIC-WOZ dataset. This well-established dataset includes various audio-visual materials and transcribed textual interactions, with participants being assessed using the Patient Health Questionnaire-8 (PHQ-8), allowing for comprehensive comparisons between individuals with depressive symptoms and those without.

By leveraging the proprietary MMD2023 dataset and the publicly available DAIC-WOZ dataset, we ensured a comprehensive and reliable evaluation of our Sentence-level multi-modal Feature Learning (SMFL) method for depression identification.

The demographic composition of the patient cohort in the MMD2023 dataset is systematically detailed in **Table 1**, ensuring fair representation in terms of age and gender distributions. This careful selection and curation of data are crucial for the development and validation of our SMFL method, which aims to achieve meticulous detection and classification of depressive episodes, consistent with the PHQ-8 gold standard, and enhance the diagnostic tools of mental health professionals.

**Table 1:** Gender and age distribution statistics of depressed patients and non-depressed individuals.

Group	Gender	Elderly	Middle aged	Young Adults	Minors	Total
Depressed Patients	Male	0	5	11	1	17
	Female	3	18	28	1	50
Non-depressed Individuals	Male	1	2	4	1	8
	Female	0	7	13	5	25
Total		4	32	56	8	100

Age groups were categorized according to international standard, with individuals aged 60 and above classified as elderly, 40 to 60 as middle-aged, 18 to 39 as young adults, and below 18 as minors. As shown in **Table 1**, the young adults accounts for 56% of the participants, and the elderly patients constitute 4% in our data. The ratios of the other age groups were 32%, and 8%, respectively in middle aged and minors. The depression was assessed by using the PHQ-8 assessment scale, with a cutoff score of 9 or above. To minimize differences in language ability, only the participants with Chinese as their native language were enrolled into the study.

The criteria for participant inclusion are as follows:

1. Outpatient patients with clear language expression and no speech difficulties. Gender is not restricted.
2. Patients are required to sign an informed consent form.
3. HAMD-17 score > 17 or PHQ-8 score ≥ 9.
4. Absence of severe suicidal risk and no conditions such as facial paralysis, disfigurement, abnormal facial movements, facial spasms, facial stroke, facial implants, vitiligo, eye diseases, speech disorders, stuttering, vocal cord surgery, or other diseases.

Exclusion criteria include:

1. Exclusion of other mental disorders and mental disorders caused by physical illnesses, substance abuse, or personality disorders.
2. Absence of psychotic symptoms during the depressive episode.
3. No history of alcohol or substance dependence, as well as no history of acute intoxication.

### 2.2 Data preprocessing

This research leverages two principal datasets, e.g. the self-collected MMD2023 and a public available DAIC-WOZ, to facilitate a robust analysis of depression indicators. The MMD2023 dataset is already described above, and DAIC-WOZ dataset comprises 192 patient cases, including 74 identified as having depressive symptoms. To ensure methodological rigor in model training and validation, both datasets were divided using an 8:2 split ratio for training and testing purposes, respectively. This structured division guarantees a balanced representation of depressive cases across both sets, thereby enhancing the reliability and validity of the study outcomes.

Especially for the MMD2023 dataset, the allocation resulted in 80 cases designated for the training set, which included 54 depressed patients. And 20 cases allocated to the testing set, with 13 depressed cases. To address the notable imbalance between depressed and non-depressed cases, the study implemented weighted ratios. These ratios were calculated based on the disparity in case quantities to ensure equitable representation and to enhance the analytical robustness of the findings. Specifically, weight calculation was performed using Formulas 1 and 2:


(1) $$proportion=[\frac{N_{0}}{S_{t}},\frac{N_{1}}{S_{t}}]$$

(2) $$weight=\frac{1}{proportion}$$

Formula 1 is the formula for calculating the proportion of positive and negative in the training data set, Formula 2 is the weight formula, $S_{t}$ represents the total number of samples in the training set; $N_{0}$ represents the number of negative samples in the training set; $N_{1}$ represents the number of positive samples in the training set.

To protect patient privacy, this study has implemented a series of measures to protect patient privacy. Firstly, personal sensitive information such as names, dates, and locations has been removed from the recorded audio and transcribed text. Secondly, in the case of video data, only the 3D facial landmarks containing 68 pixels, which are essential for measuring facial movements such as eye, lip, and head movements, have been retained. This ensures that there is not enough information available to identify individuals, only sufficient information for measuring facial movements.

### 2.3 Feature extraction for each modal

This section elaborates the feature learning methods for each modality (video, audio and text) utilized by the proposed SMFL model.

#### 2.3.1 Video Features

To navigate the communicative challenges between depressed patients and doctors, physicians often rely on facial muscle changes and eye movements to gauge psychological states. These nuances are quantified as Action Units (AUs) by Ekman et al. (19). AUs reflect the synchronized activities of facial muscle groups during specific expressions and have been effectively utilized in multiple studies for automated depression assessment, showing promising outcomes. Additionally, visual features such as facial key points, eye gaze direction, and head posture are also employed in evaluating depression. For detailed information, please refer to **Supplementary Table S1**.

#### 2.3.2 Audio Features

Empirical studies indicate that speech segments from depressed individuals often display prolonged pauses and muddled expressions (20). Disturbances in rhythm have also been observed in patients with mental disorders (21). In response, this study incorporates auditory data encompassing these characteristics as key input features. Notably, eight frequency-related parameters are included, such as the fundamental frequencies of the first, second, and third formants, jitter, frequency, and bandwidth. Furthermore, three amplitude-related parameters—loudness, shimmer, and the harmonic-to-noise ratio—are analyzed. Additionally, 14 spectral parameters, including the alpha ratio and MFCCs, are utilized. For detailed information, please refer to **Supplementary Table S2**.

#### 2.3.3 Text Features

The semantic content of patient responses is instrumental in assessing depressive states (22). Often, text data from patients exhibit a strong emotional undertone, with statements such as “I’ve ruined my family, they would be better off without me” or “I’m worthless.” In this study, an enhanced version of the EmoBertA (23) model is employed to extract pertinent text features, facilitating the differentiation of depression-related text. For detailed information, please refer to **Supplementary Table S3**.

### 2.4 Model description

**Figure 1:**
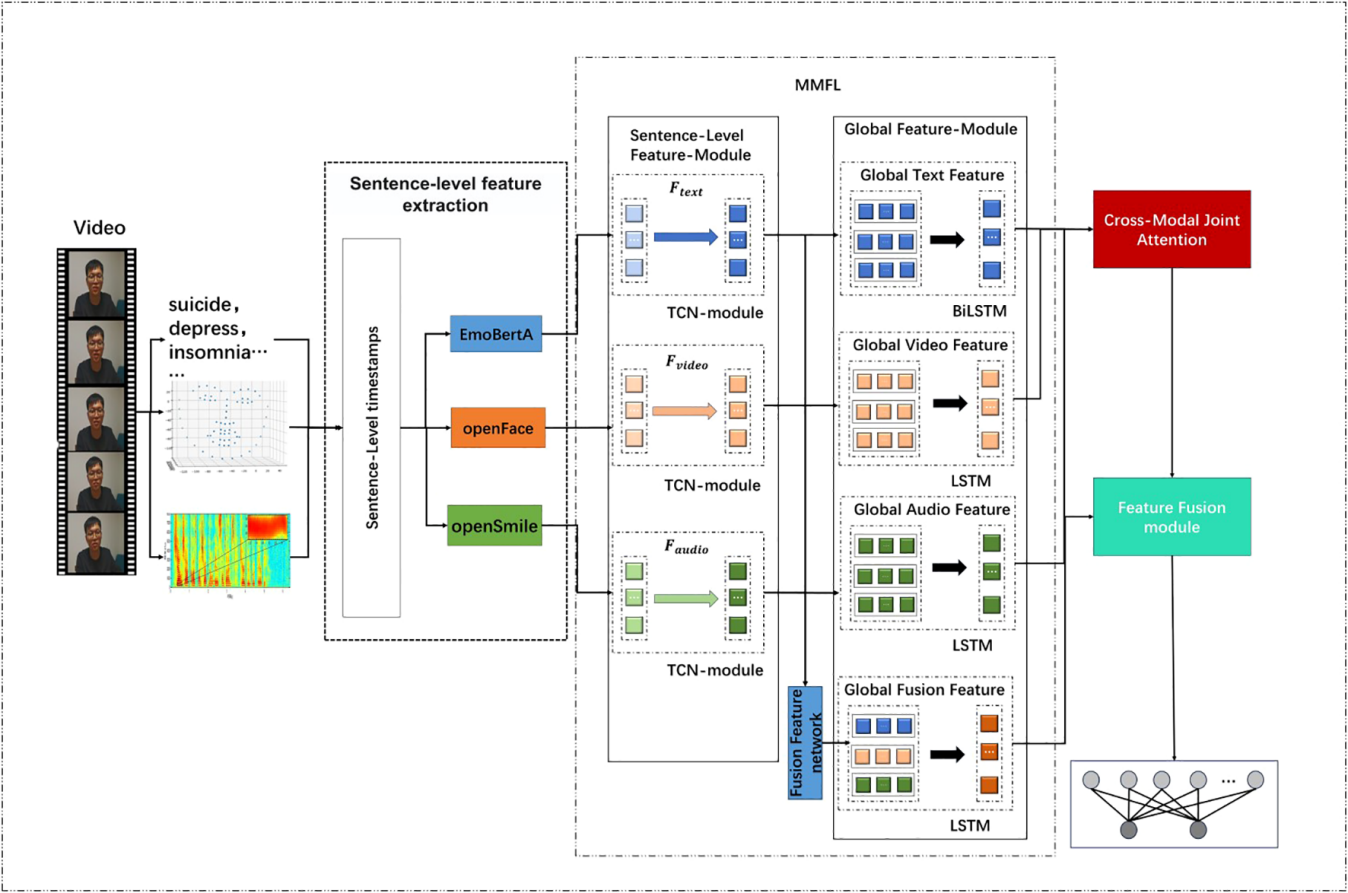
The proposed SMFL architecture.

The proposed model, SMFL (Sentence-level Multi-modal Feature Learning), consists of four modules: sentence-granularity feature extraction, Multi-modal Feature Learning (MMFL), Cross-Modal Joint Attention (CMJAT), and the Feature Fusion module. The overall architecture of the model is illustrated in **Figure 1**.

SMFL utilizes three modalities as inputs: temporal facial features, patient audio features, and interview transcript text features. In the sentence-granularity feature extraction module, the patient video is segmented based on sentence-level timestamps, and the corresponding encoder is used to extract sentence-level features for each modality. To enhance feature representation, the extracted features from each modality are fed into the MMFL module. Meanwhile, the MMFL module employs a Fusion Feature network to integrate and process features from different modalities. Specifically, the Fusion Feature network receives features from audio, video, and text modalities, and by merging and processing these features, it generates a global fused feature $F_{fusion}$ , providing a comprehensive view and rich semantic information. The LSTM module in MMFL effectively models the contextual relationships of the sequences, which is beneficial for depression detection tasks.

Additionally, to reduce model complexity and improve efficiency, the MMFL module introduces a variable threshold value, *M*, to represent the upper limit of the number of sentences. The CMJAT module establishes connections between the video and text modalities, strengthening the facial features. After obtaining the multi-modal sentence-level features, they are passed to the global feature extraction unit.

Finally, Feature Fusion module is performed using Cross-Attention, and the final depression recognition label is obtained through a fully connected layer. This sophisticated approach ensures that the model captures the nuanced interactions between different modalities, thereby improving the accuracy and reliability of depression detection.

### 2.5 Multi-modal feature learning

**Figure 2:**
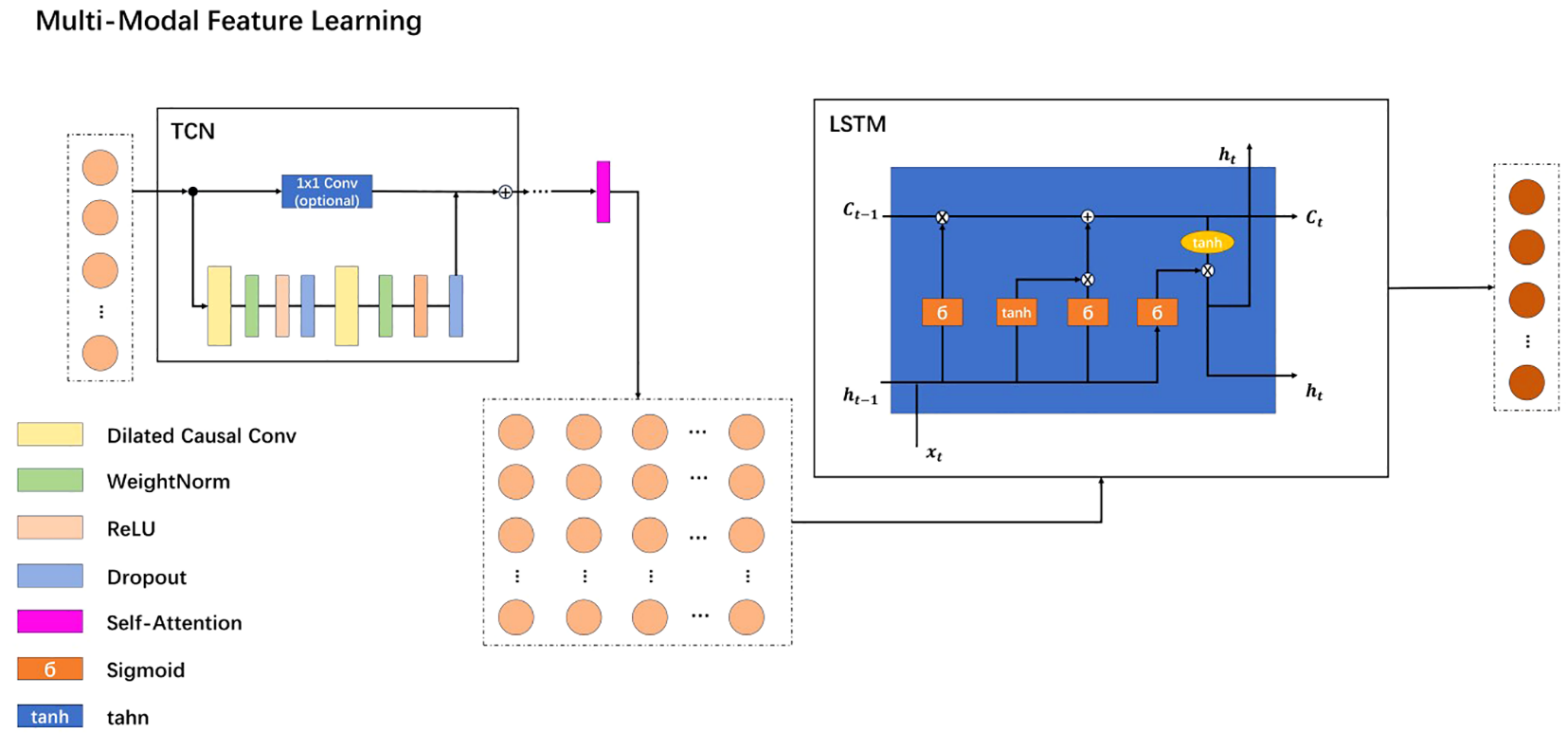
MMFL model architecture.

The enhanced feature representation of patient data is significantly advanced by the Multi-Modal Feature Learning (MMFL) framework, as illustrated in **Figure 2**. The process begins with sentence-level features being fed into a Temporal Convolutional Network (TCN) module. To augment the efficacy of the TCN, this study integrates causal convolutions, dilated convolutions, and residual blocks. These elements are strategically designed to modulate the receptive field size during temporal feature extraction, ensuring that parameter count remains manageable and system complexity does not become prohibitive.

After completing the initial TCN processing, a self-attention mechanism is inserted. This self-attention mechanism processes sentence-level features at this stage to better capture long-term dependencies and semantic associations between sentences. Such an insertion can further enhance the model’s representation capabilities for sentence-level features, improving performance in tasks like depression recognition. Subsequently, the sentence-level features are aggregated into global features and then passed as inputs to the Long Short-Term Memory (LSTM) model. The LSTM module has several advantages over the TCN configuration.

Firstly, through its complex memory cells and gating mechanisms, LSTM can flexibly capture and retain long-term dependencies in data sequences. This ability makes LSTM excel at recognizing long-range temporal dependencies, whereas TCN’s reliance on convolutional strategies may encounter issues.

Furthermore, LSTM addresses the common neural network challenges of vanishing and exploding gradients, thereby enhancing stability during the training of deep networks and improving its ability to discern complex patterns within time-sequence data.

By synergistically combining TCN and LSTM, the MMFL framework leverages the strengths of both approaches, including efficient temporal feature extraction and comprehensive management of model complexity while effectively capturing long-range dependencies and mitigating gradient-related challenges. This dual-approach architecture positions the proposed model as a particularly promising tool for improving the feature representation of patient data, thereby enhancing diagnostic capabilities in the context of depression recognition.

### 2.6 Cross-modal joint attention

**Figure 3:**
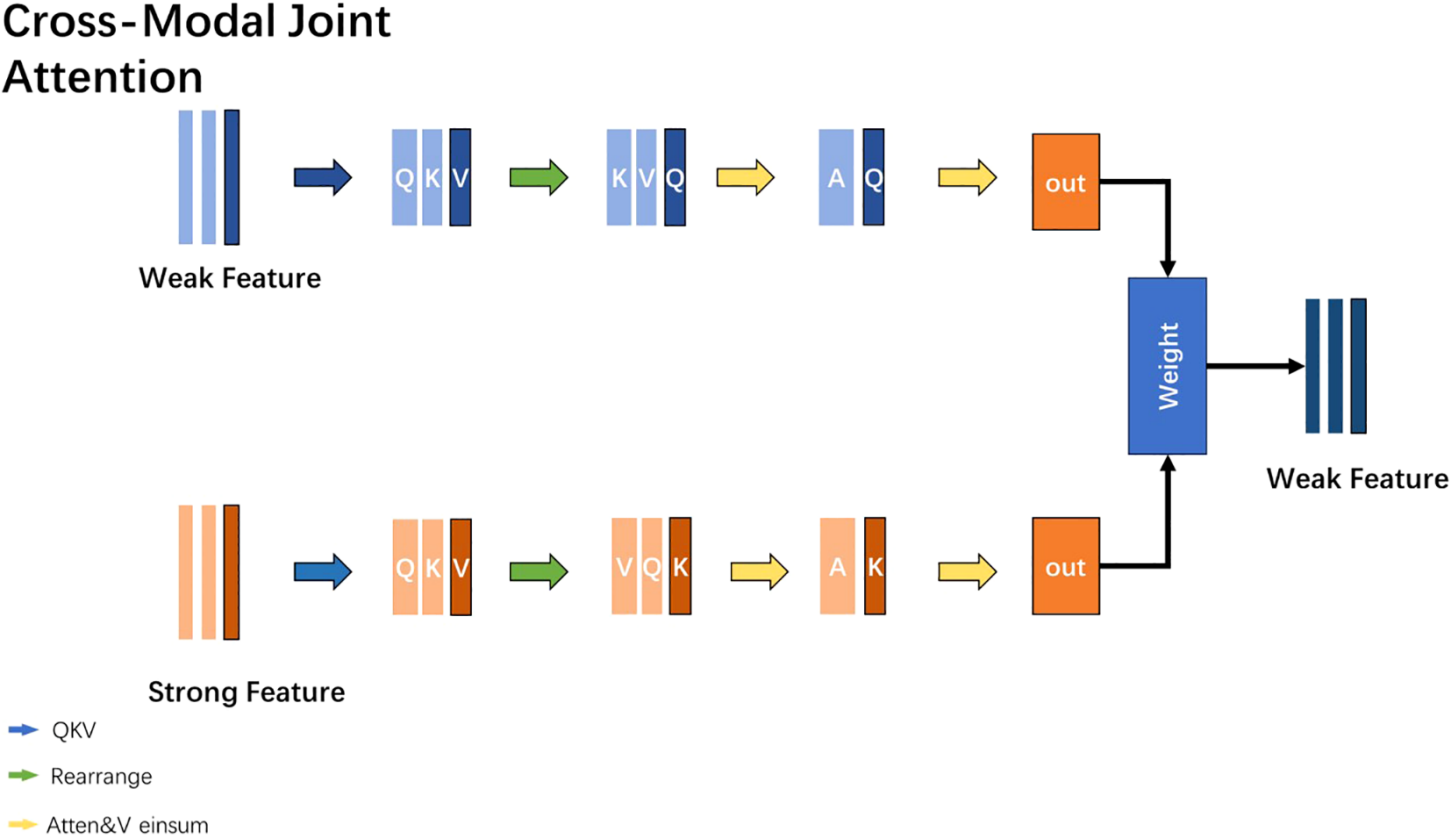
Enhancing weak features using CMJAT.

During the research process, it was observed that multi-modal data often exhibits a distinction between strong and weak modalities (text and video, respectively). Based on this phenomenon, this study introduces a Cross-Modal Joint Attention (CMJAT) mechanism, whose structure is depicted in **Figure 3**. The core idea of CMJAT is to amplify weakly expressed features and enhance their impact during model training, thereby boosting the model’s capability in depression recognition. This mechanism utilizes an attention framework to concentrate the model’s focus on two closely related features during training.

Specifically, CMJAT begins by calculating the similarity between the features of strong and weak modalities. It then adjusts the importance of each modality feature using attention weights. The aim is to shift the model’s focus towards task-relevant and expressive features, simultaneously enhancing the influence of weaker features. By integrating CMJAT, the model effectively leverages strong features to bolster the expressive power of weak features. This approach balances the significance of different features in multi-modal data, thereby enhancing the overall performance of the model in recognizing depression.

The steps of the CMJAT mechanism are shown as follows:

#### 2.6.1 Feature projection

The features from different modalities are transformed into three-dimensional Query, Key, and Value vectors using linear layers. This transformation allows for subsequent operations. The formulas are as follows (Equations 3, 4, and 5):


(3) $$Q_{out}=F_{input}\cdot WQ_{weight}+bQ_{bias}$$

(4) $$K_{out}=F_{input}\cdot WK_{weight}+bK_{bias}$$

(5) $$V_{out}=F_{input}\cdot WV_{weight}+bV_{bias}$$

In the given formula, $F_{input}$ represents the input feature data, and $WQ_{weight}$ , $WK_{weight}$ , $WV_{weight}$ are weight matrices used to project the data into the Query, Key, and Value vector spaces, respectively. $bQ_{bias}$ , $bK_{bias}$ , $bV_{bias}$ represent the bias vectors.

#### 2.6.2 Random permutation

To further enhance the model’s generalization ability and robustness, the QKV data obtained from different modalities is randomly permuted. This serves as a data augmentation technique. By randomly shuffling the order of the QKV vectors, the model is exposed to different permutations of the multi-modal features, thus improving its ability to handle various input configurations. This helps the model generalize better to unseen data and enhances its robustness to variations in feature ordering.

#### 2.6.3 Using the einsum function

The next step is to calculate the similarity values between the Query and Key vectors of each modality using the einsum function. This operation allows for efficient computation of the similarity scores. The formula is as follows (Equation 6):


(6) $$sim=einsum\left('b\;i\;d,b\;j\;d\to b\;i\;j^{\prime },q,k\right)$$

where q is the query tensor with a shape of (batch_size, num_queries, query_dim), *k* is the key tensor with a shape of (batch_size, num_keys, key_dim). The einsum function is used to calculate the similarity between the queries (*q*) and keys (*k*). This will generate a tensor sim with a shape of (batch_size, num_queries, num_keys). Then, the softmax function is applied to compute the weights of the tensor *sim*. Finally, using the einsum function again, the similarity between sim and the value tensor v is calculated to obtain the weighted output tensor out with a shape of (batch_size, num_queries, value_dim).

#### 2.6.4 Adjusting weights based on the results

In the domain of depression diagnosis, evaluation is frequently carried out using screening tools such as the Patient Health Questionnaire-8 (PHQ-8), which assigns scores to patients’ responses to ascertain the presence of depressive symptoms. In the Cross-Modal Joint Attention (CMJAT) mechanism, textual and video features are concatenated, followed by the application of a multi-head self-attention mechanism between them. The model then takes the enhanced video features as input and channels them through a dense (fully connected) layer to make predictions regarding depression. Subsequent to this process, the results are analyzed, and the parameters of the Weight layer are fine-tuned accordingly. This iterative optimization is aimed at refining the model’s accuracy and robustness in detecting depression.

### 2.7 Feature fusion module

In the Feature Fusion module, the Cross-Attention (CrossATT) mechanism is used to compute the relationship mapping between the global fused feature $F_{fuse}$ and the global text feature $F_{text}$ , global audio feature $F_{audio}$ and the video feature $F_{Video}^{CMJAT}$ enhanced through CMJAT, The normalized modality mappings are multiplied element-wise with $F_{fuse}$ , resulting in the CrossATT features $F_{\text{text}}^{\text{CA}}$ , $F_{\text{Video}}^{\text{CA+CMJAT}}$ and $F_{\text{audio}}^{\text{CA}}$ . Finally, the original $F_{fuse}$ feature and the CrossATT features are concatenated.

**Figure 4:**
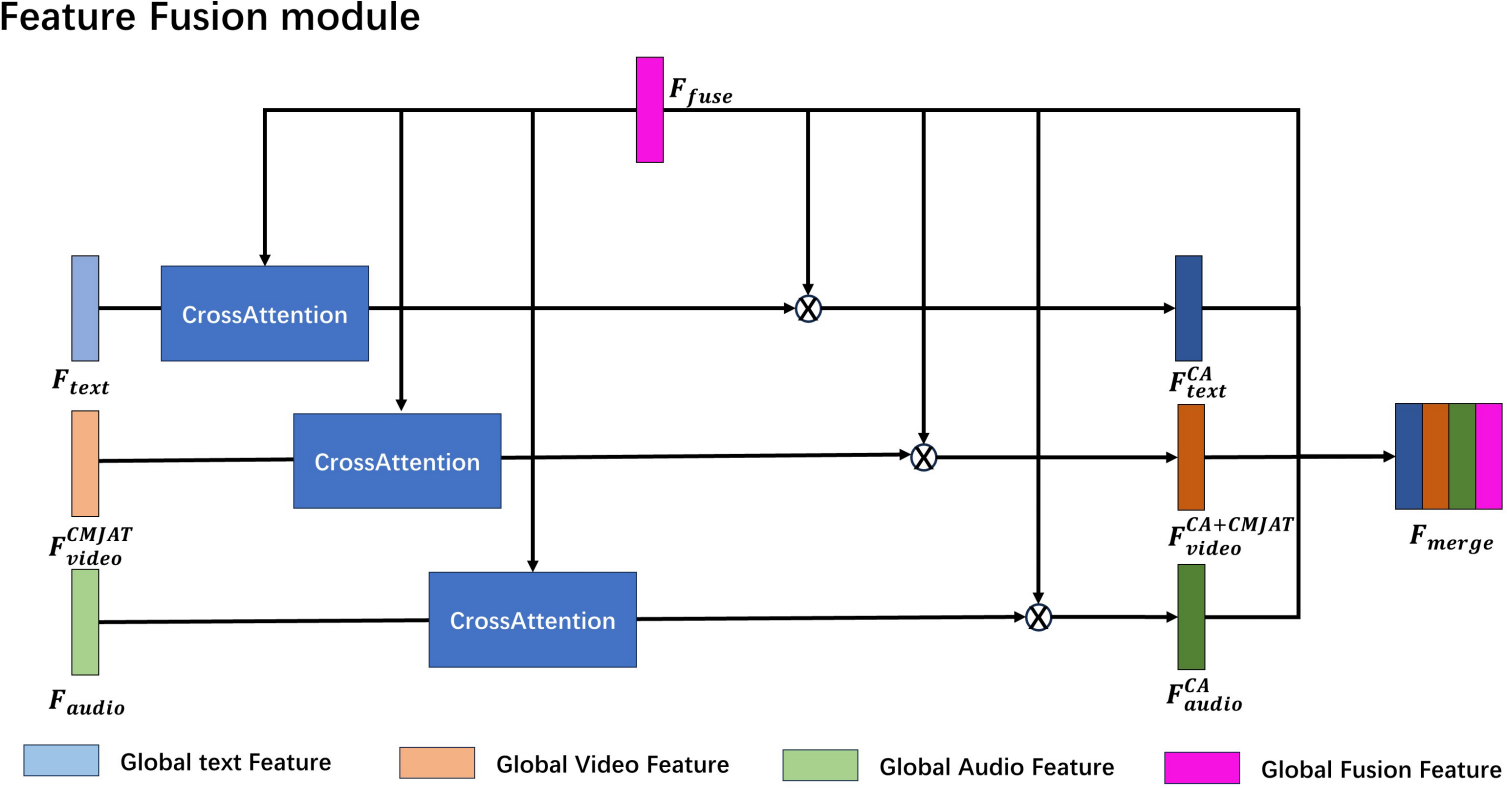
Feature-Fusion model architecture.

The structure of this process is depicted in **Figure 4**. The purpose of this approach is to address potential issues in global feature fusion during multi-modal model training, where certain modality features may dominate and overshadow others. By introducing the CrossATT mechanism, the relationships between different modalities can be modeled more accurately, thereby enhancing the expressive capacity of multi-modal features. This feature fusion method effectively leverages the complementarity between different modalities, thereby improving the overall performance and robustness of the model.

### 2.8 Model analysis

The features within depression datasets are inherently complex and multi-modal. Consequently, it is common to encounter scenarios where the feature dimensions are potentially larger than the sample size. Such data characteristics frequently precipitate overfitting issues in prevailing deep learning-based feature extractors. To resolve this issue, our proposed SMFL method separately analyzes data from multiple modalities, thereby accurately identifying depression patients from high-dimensional features with only a small number of samples needed.

Specifically, the SMFL model initially aggregates multi-modal data encompassing facial expressions, vocal attributes, and transcribed texts, thereby effectively fortifying the model’s robustness and mitigating the bias induced by single-modality data. Subsequently, the model leverages TCN and LSTM networks to meticulously mine the long-term dependencies within each modality, thus enhancing the model’s generalization capability even on small-scale datasets. Finally, the model incorporates the CMJAT mechanism, which is adept at harmonizing and balancing the relationships among different modalities, ensuring a comprehensive capture of depressive symptoms. By integrating these components, our SMFL model mitigates the overfitting issues that arise when the feature dimensionality exceeds the sample size, thereby accurately distinguishing patients with depression.

## 3 Results

### 3.1 Evaluation metrics

In this study, precision, recall, specificity, F1-score, and accuracy were used as evaluation metrics to assess the performance of different classification models. Precision measures the proportion of true positive predictions out of all predicted positive instances. Its formula is as follows (Equation 7):


(7) $$Precision=\;\frac{TP}{TP+FP}$$

Recall, on the other hand, measures the proportion of true positive predictions out of all actual positive instances (24). In an ideal scenario, higher precision and recall indicate better model performance. However, there is often a trade-off between precision and recall. The formula for recall is as follows (Equation 8):


(8) $$recall=\;\frac{TP}{TP+FN}$$

F1-score serves as the harmonic mean of precision and recall, providing an overall assessment of the model’s performance on these two metrics (25). Additionally, specificity is another important metric that measures the proportion of true negative predictions out of all actual negative instances. A higher specificity indicates a lower rate of false positives. The formulas for specificity are as follows (Equations 9 and 10):


(9) $$F1-score=\;\frac{2TP}{2TP+FP+FN}$$

(10) $$Specificity=\;\frac{TN}{TN+FP}$$

Lastly, accuracy is used to measure the proportion of correctly classified instances out of all samples in a classification model. It is a commonly used evaluation metric that provides an understanding of the overall classification performance of the model. The formula for accuracy is as follows (Equation 11):


(11) $$Accuracy=\;\frac{TN+TP}{TP+TN+FP+FN}$$

In the above formulas: TP (True Positives) represents the number of positive samples correctly classified. FP (False Positives) represents the number of negative samples incorrectly classified as positive.TN (True Negatives) represents the number of negative samples correctly classified. FN (False Negatives) represents the number of positive samples incorrectly classified as negative.

The issue of data imbalance frequently arises in depression datasets, potentially skewing model performance metrics. To address this, we primarily employ Accuracy for primary assessment, providing an initial gauge of the model’s overall performance. Additionally, we incorporate Precision, Recall, and Specificity to facilitate a more comprehensive evaluation. Furthermore, we employ the F1-score, which harmonizes Precision and Recall into a single metric, offering a nuanced assessment of the model’s performance in the context of imbalanced data. This multi-faceted evaluation strategy ensures a robust and nuanced understanding of the model’s efficacy in identifying depression.

### 3.2 Detailed description of implementation

The experiments in this study were conducted on a Linux operating system, utilizing hardware resources primarily consisting of eight GeForce RTX 3080Ti GPUs. The software environment included Python 3.6.6 and the PyTorch 1.7.0 framework. The baseline network employed for the experiments was the Temporal Convolutional Network (TCN), which was further optimized to obtain the Sentence-level Multi-modal Feature Learning (SMFL) model. The key parameters of the SMFL model were set as follows: an initial learning rate of 1e-3, Adam optimizer, 5 hidden layers, and 12 hidden channels in the sentence-level feature extraction, a convolutional kernel size of 5, and a dropout rate of 0.5. Each experiment was conducted for a maximum of 100 epochs, with the learning rate reduced by a factor of 10 every 5 epochs. If the loss on the test set did not decrease for 10 consecutive epochs, the training process was stopped. Upon evaluation with the fully trained SMFL model, the inference time for a single sample was approximately 5.479 second. The code for the proposed SMFL methodology has been made publicly accessible at https://github.com/Crisp1031/SMFL.

### 3.3 Experimental results

#### 3.3.1 Comparative experiments on different dataset

To reveal the effectiveness of our SMFL approach, a comparative evaluation was conducted on our self-collected MMD2023 dataset (**Table 2**) and the publicly available DAIC-WOZ dataset (**Table 3**).

**Table 2:** The depression recognition results of our SMFL on MMD2023 dataset.

Model	F1-score	Specificity	Precision	Recall	Accuracy
TCN (all)	0.79 ± 0.04	0.79 ± 0.04	0.81 ± 0.03	0.79 ± 0.05	0.81 ± 0.05
TCN (sentence)	0.83 ± 0.04	0.79 ± 0.04	0.87 ± 0.03	0.79 ± 0.05	0.85 ± 0.05
TCN + Self-Attention + Cross-Attention	0.85 ± 0.03	0.84 ± 0.03	0.88 ± 0.03	0.84 ± 0.03	0.86 ± 0.03
LSTM + Self-Attention + Cross-Attention	0.91 ± 0.03	0.84 ± 0.03	0.93 ± 0.03	0.84 ± 0.03	0.90 ± 0.03
SMFLM	0.93 ± 0.03	0.90 ± 0.03	0.94 ± 0.03	0.88 ± 0.03	0.91 ± 0.03

**Table 3:** The depression recognition results of our SMFL on DAIC-WOZ dataset.

Model	F1-score	Specificity	Precision	Recall	Accuracy
TCN (all)	0.76 ± 0.04	0.78 ± 0.04	0.71 ± 0.03	0.83 ± 0.05	0.77 ± 0.05
TCN (sentence)	0.79 ± 0.04	0.81 ± 0.04	0.74 ± 0.03	0.81 ± 0.04	0.77 ± 0.05
LSTM + Self-Attention	0.81 ± 0.04	0.83 ± 0.03	0.80 ± 0.03	0.84 ± 0.05	0.82 ± 0.04
LSTM + Self-Attention + Cross-Attention	0.83 ± 0.04	0.81 ± 0.05	0.80 ± 0.03	0.85 ± 0.05	0.83 ± 0.04
TCN + Self-Attention	0.81 ± 0.05	0.79 ± 0.05	0.80 ± 0.03	0.80 ± 0.03	0.80 ± 0.05
TCN + LSTM + Self-Attention	0.84 ± 0.05	0.80 ± 0.04	0.78 ± 0.03	0.83 ± 0.04	0.81 ± 0.05
Zou et al. (20)	0.87	0.79	0.94	0.81	0.86
C-CNN (12)	0.77	0.56	0.71	0.83	0.70
**SMFLM**	**0.89** ± **0.03**	**0.85** ± **0.03**	**0.94** ± **0.04**	**0.84** ± **0.05**	**0.89** ± **0.04**

The bold text is the best result among each column.

##### 3.3.1.1 Result on self-collected clinical data

In **Table 2**, the experimental results are based on the self-collected dataset MMD2023. It can be observed that the proposed SMFL model outperforms other models, achieving F1-score, specificity, precision, recall, and accuracy of 93%, 90%, 94%, 88%, and 91%, respectively. This indicates that the model exhibits excellent performance and generalization ability in Chinese depression recognition tasks, being able to adapt to the characteristics of different depression datasets. Comparing TCN (all) and TCN (sentence), it can be observed that using sentence-level data outperforms directly using complete segment data. Specifically, incorporating sentence-level data leads to improvements of 4% in F1-score, 6% in precision, and 4% in accuracy.

Furthermore, combining TCN (sentence) with Self-Attention and Cross-Attention mechanisms further enhances the model’s performance, confirming the utility of these attention mechanisms in depression recognition. After incorporating the attention mechanisms, there are additional improvements of 2% in F1-score, 5% in specificity, 1% in precision, 5% in recall, and 1% in accuracy. Replacing TCN (sentence) with LSTM results in a 6% increase in F1-score, a 5% increase in precision, and a 4% increase in accuracy. This suggests that using the LSTM model slightly outperforms the TCN model in data processing. Finally, the proposed SMFL model demonstrates superior performance compared to other models.

**Figure 5:**
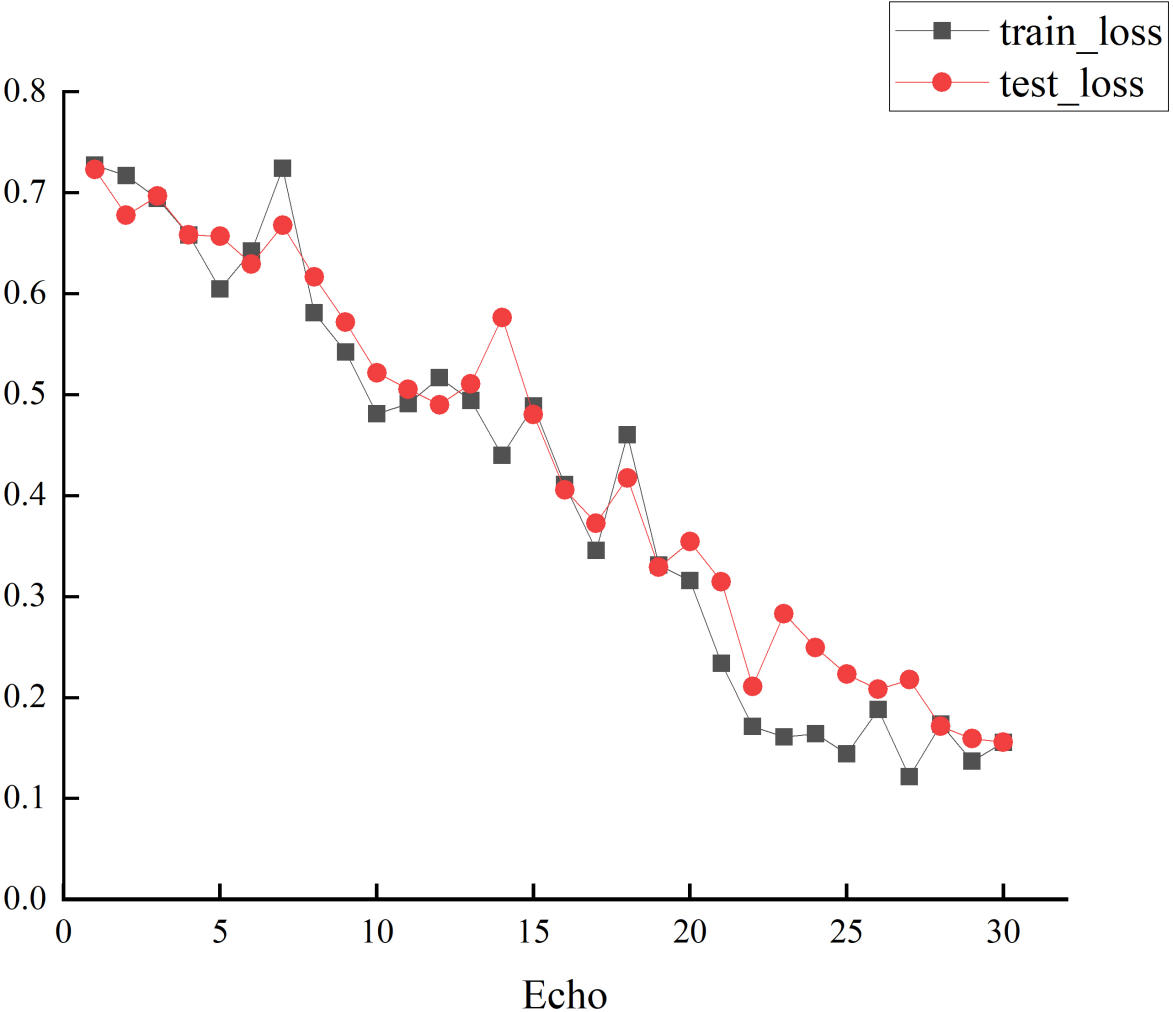
The TrainLoss and TestLoss values in this study.

Compared to TCN (all), the SMFL model achieves improvements of 14% in F1-score, 11% in specificity, 13% in precision, 7% in recall, and 10% in accuracy. The loss values of this experiment are shown in **Figure 5**. Additionally, this study validates the feasibility of the visual feature extraction method used in the data preprocessing stage, as well as the effectiveness of feature parameter selection. These findings highlight the SMFL model as a more effective choice for depression recognition in handling multi-modal data.

In summary, the SMFL model performs remarkably well on real-world clinical data, effectively addressing practical issues and demonstrating significant clinical applicability. It provides powerful decision support for clinical practitioners and holds potential for application in actual depression diagnosis and treatment. By integrating multi-modal data and leveraging advanced deep learning techniques, the SMFL model can offer more accurate and reliable depression recognition results, significantly improving the diagnosis and treatment of depression patients. Therefore, the SMFL model holds tremendous potential for wide-ranging clinical applications.

##### 3.3.1.2 Results on clinical data

To evaluate the generalization ability of the proposed SMFL model, we also conducted the experiments on the public dataset DAIC-WOZ. The experimental results are summarized in **Table 3**. The SMFL model achieved outstanding results on the public dataset, with F1-score, specificity, precision, recall, and accuracy reaching 89%, 85%, 94%, 84%, and 89%, respectively. Comparing the experiments using TCN (all) and TCN (sentence), it can be noticed that using sentence-level data performs better than directly using complete segment data, both on the private and public datasets. In these experiments, the F1-score, specificity, and precision improved by 3%. Comparing TCN (sentence) with TCN + Self-Attention, it can be seen that adding attention mechanism led to improvements of 2% in F1-score, 6% in precision, and 3% in accuracy.

The experiments comparing LSTM + Self-Attention and TCN + Self-Attention demonstrate that replacing the model with LSTM significantly improves the performance of multi-modal depression recognition. This indicates that using memory-based neural network algorithms enhances the potential for recognition. Specifically, specificity, recall, and accuracy improved by 4%, 4%, and 2% respectively. According to the results of LSTM +Self-Attention and LSTM + Self-Attention + Cross-Attention experiments, it can be concluded that using CrossATT for global feature processing on top of self-attention leads to better results. This demonstrates the effective utilization of complementary modalities by integrating CrossATT. The experiments involving TCN + Self-Attention and TCN + LSTM + Self-Attention, which utilize LSTM models for global feature processing, show that compared to TCN, LSTM models can effectively capture and maintain long-term temporal dependencies. This validates the practicality of the proposed SMFL framework. In conclusion, through comparative experiments, the SMFL model has demonstrated its high effectiveness in integrating LSTM algorithm and CMJAT into a sentence-level TCN model for multi-modal depression recognition, outperforming the models proposed by Zou et al. (20)and C-CNN (4, 12). The model significantly reduces the misdiagnosis rate and provides valuable assistance in diagnosing depression.

In summary, the proposed SMFL model also exhibits excellent performance on the publicly available DAIC-WOZ dataset, indicating strong generalization, robustness, and stability. By integrating sentence-level data, attention mechanisms, LSTM models, and CMJAT, the SMFL model achieves significant improvements in multi-modal depression recognition. These research findings emphasize the model’s applicability in different datasets and scenarios, providing a valuable tool and approach for assisting in depression diagnosis. These discoveries have important implications for future depression research and clinical practice.

#### 3.3.2 Ablation

To further validate the superiority of the proposed model and the rationale behind the incorporated components, we conducted ablation experiments on the publicly recognized DAIC-WOZ dataset. These experiments assessed the model’s performance with the inclusion of various components, while keeping other hyperparameters constant.

##### 3.3.2.1 Evaluation of strong and weak feature representations

To evaluate the impact of different modal features on the model, two experiments, including evaluation the of the different unimodal feature and combination methods, were conducted on the DAIC-WOZ dataset.

**Figure 6:**
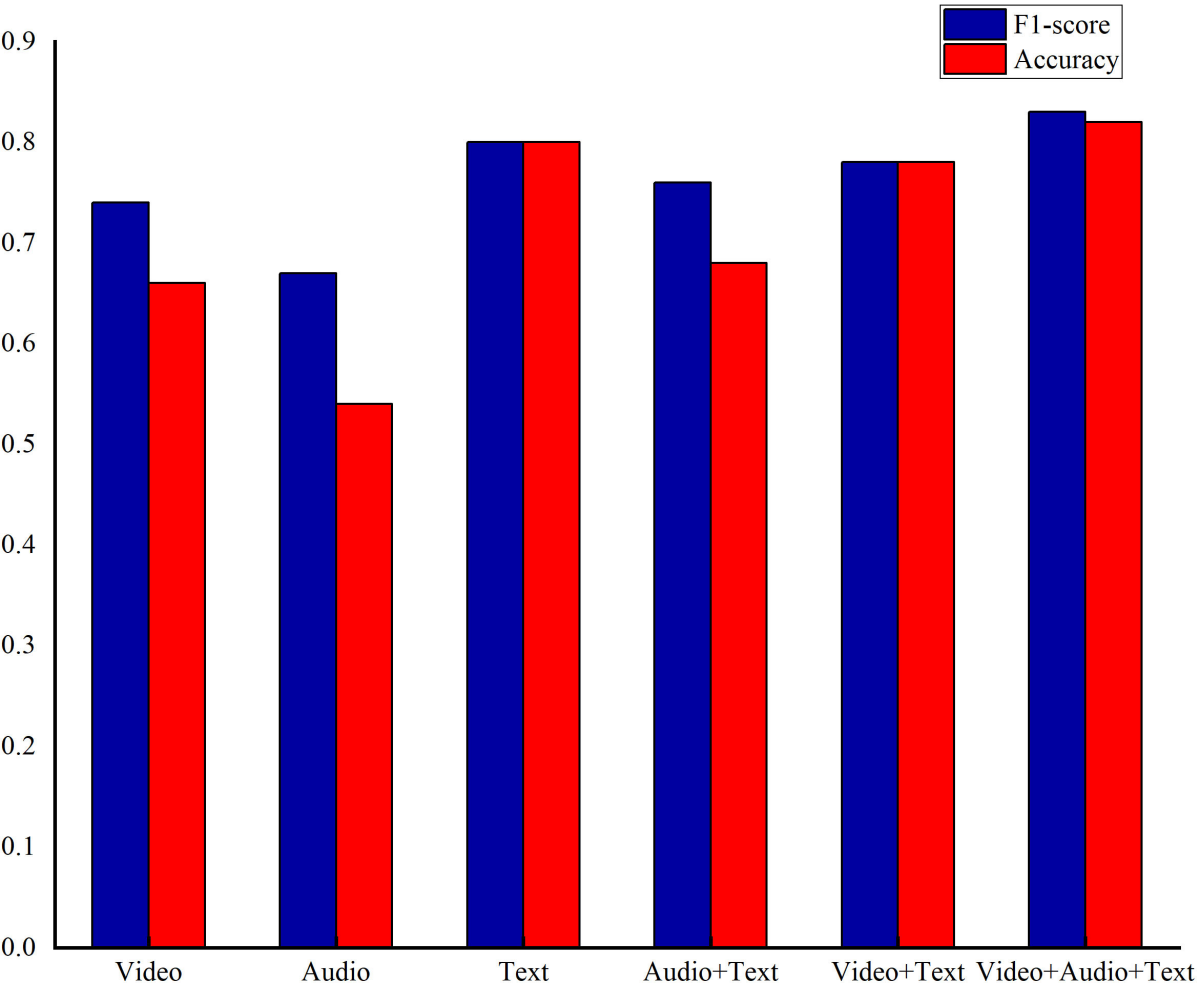
The results of depression recognition using different single-modal data and enhanced single-modal features with Cross-Modal Joint Attention under the base model.

In the evaluation of different unimodal features, a unimodal model was employed with video features, audio features, and text features considered as separate inputs. The TCN-based model served as the reference for comparison. When assessing various multi-modal feature combinations, the CMJAT (Cross-Modal Joint Attention) mechanism was utilized to weigh the features. Video and audio features were categorized as weak features, while transcribed text features were considered strong features. The base model was used for comparison, and the experimental results are detailed in **Figure 6**.

From **Figure 6**, the text modal achieves best performance on F1-score and accuracy among unimodal settings, while the video and audio perform relative weak performance. As a result, text features were leveraged as strong features to reinforce the video features, aiming to enhance the model’s accuracy, while others are treated as weak ones in the attention module. Furthermore, the feature fusion among video, audio, and text performs the best results, demonstrating the superiority of our proposed multi-model feature fusion approach.

##### 3.3.2.2 The effectiveness of feature extraction

This part evaluates the influence of different feature extractors on our proposed SMFL model, which results are shown in **Table 4**.

**Table 4:** Experimental results of different feature extractors and attention mechanisms in depression recognition.

Model	F1-score	Specificity	Precision	Recall	Accuracy
TCN (all)	0.76	0.78	0.72	0.83	0.77
TCN (sentence)	0.79	0.81	0.75	0.85	0.79
TCN (sentence) + LSTM	0.83	0. 81	0.82	0.85	0.82
MMFL	0.83	0.80	0.83	0.80	0.82
MMFL + Cross-Attention	0.85	0.83	0.87	0.90	0.85
**MMFL + Cross-Attention +CMJAT**	**0.89**	**0.85**	**0.93**	**0.85**	**0.89**

The **bold** text is the best result among each column.

To evaluate the impact of sentence-level structure on model performance, the SMFL was modified to a sentence-level TCN model. The results demonstrated a positive effect of using sentence-level data as input for depression recognition tasks. Subsequently, in further experiments, the LSTM model was incorporated into the sentence-level TCN model to handle subsequent global features. The results showed a 4% improvement in F1-score, a 7% improvement in precision, and a 3% improvement in accuracy, validating the effectiveness of the LSTM component in enhancing model performance.

Based on the aforementioned experimental results, it is evident that the proposed SMFL model exhibits significant performance improvements depression recognition task. This signifies the ability of SMFL to effectively enhance performance metrics, including F1-score, precision, and accuracy, in multi-modal depression recognition. Furthermore, these findings further affirm the superiority and potential of the SMFL model in handling depression recognition task.

##### 3.3.2.3 Attention mechanism

In this section, different attention mechanisms were added to SMFL model, and the results are as follows. It was observed that utilizing the MMFL framework, which involves inserting a self-attention mechanism between the TCN and LSTM modules, further enhanced the model’s performance. Subsequently, Cross-Attention was introduced on top of MMFL framework to fuse subsequent features. Compared to Self-Attention alone, the evaluation metrics of the model improved by 2%, 3%, 4%, 10%, and 3%, respectively, confirming the effectiveness of integrating Cross-Attention into the model. Finally, the Cross-Modal Joint Attention mechanism (CMJAT) was added to amplify the representation of weak features. Compared to Cross-Attention, there were improvements of 4% in F1-score, 2% in specificity, 6% in recall, and 4% in accuracy. Furthermore, when compared to the baseline TCN model, the overall performance of the model was significantly optimized, with improvements of 13%, 7%, 21%, 2%, and 12% in the respective metrics. Based on these experiments, it can be concluded that the components integrated into the SMFL model have demonstrated excellent performance in depression recognition tasks.

## 4 Discussion

Given the growing research in depression recognition, our study aims to overcome the challenges associated with multi-modal depression detection. Our investigation explored a variety of modalities, incorporating patient response text, speech data, and 3D facial landmarks, among others. We introduced the Sentence-level Multi-modal Feature Learning (SMFL) architecture, an innovative framework designed for effective feature extraction from patient data. Concurrently, we developed the Cross-Modal Joint Attention (CMJAT) mechanism to enhance weaker modalities by leveraging the strengths of more expressive ones.

To thoroughly evaluate the efficacy of our proposed approach, we collected a Chinese depression dataset, MMD2023 and involved a widely recognized DAIC-WOZ dataset to conduct experiments. The MMD2023 dataset, composed of multi-modal features from 100 participants (67 depressed patients and 33 non-depressed individuals), was meticulously assessed by professional psychiatrists and includes a diverse range of demographic backgrounds, which is critical for ensuring the generalizability of our findings across different cultural contexts. Besides, the DAIC-WOZ dataset, containing data from 192 patients, served as a robustness and generalization benchmark for validating our model’s performance.

Through rigorous experimentation on these diverse datasets, our results have indicated substantial enhancements over preceding models. Specifically, our SMFL approach achieved accuracies of 91% on the MMD2023 dataset and 89% on the DAIC-WOZ dataset, demonstrating its superior performance in binary depression classification. These results underscore the potential of our methodology to provide a more precise and balanced multi-modal feature representation, which is crucial for accurate depression detection.

Simultaneously, the fully trained SMFL model can accomplish depression diagnosis for an individual patient within approximately five seconds. Although this duration may not be exceptional among AI algorithms due to the comprehensive analysis of multi-modal data and the trade-off for accurate diagnostic performance, it remains significantly more efficient than traditional manual diagnosis. Moreover, the intelligent diagnostic algorithm can alleviate the workload of clinicians, aiding them in conducting depression diagnosis and treatment more efficiently.

We have identified that diagnostic algorithms will be influenced by different language habits, living environments, cultural exchange, and other related factors, leading to biases. But most extant depression datasets are predominantly in English. To leverage these biases, we are in the process of collecting the Chinese depression dataset MMD2023. This dataset stands out by featuring an expanded participant pool and multiple scales meticulously evaluated by professional psychiatrists, thereby offering a significant contribution to the domain of Chinese depression diagnosis.

Despite the demonstrable advantages of the proposed SMFL algorithm in small-scale depression classification tasks, several areas remain open for optimization. For instance, due to practical constraints, the MMD2023 dataset collected in this study comprises a limited number of samples and lacks extensive multi-class classification for the cases. Additionally, the computational complexity of the proposed model presents further opportunities for refinement. We acknowledge these limitations and are committed to addressing them in future work, thereby pushing the boundaries of multi-modal depression recognition.

Constrained by the scarcity of domestic depression datasets, our future work will primarily focus on augmenting the MMD2023 dataset by incorporating a more diverse range of participants, and introducing multi-class labels to facilitate a more comprehensive multi-class diagnosis of depression severity. Concurrently, we will undertake a rigorous optimization of the SMFL model in future to reduce computational complexity while maintaining diagnostic efficacy, thereby enhancing the overall efficiency and applicability of the model in clinical settings.

## Data Availability

The original contributions presented in the study are included in the article/**Supplementary Material**. Further inquiries can be directed to the corresponding authors.
