# Supplementary material for: Sentence-level multi-modal feature learning for depression recognition
Source: Front Psychiatry. 2025 Mar 21;16:1439577. doi: 10.3389/fpsyt.2025.1439577 (PMC11969223; doi:10.3389/fpsyt.2025.1439577)
Supplement: Supplementary file 1 [file Table1.docx]

**Table S1** Specific input facial features for the SFML model

| Feature Category | Facial Features | Feature Description |
| --- | --- | --- |
| 3D Facial Landmark Coordinates | x0, x1, x2, x3, ..., x67, y0, y1, y2, y3, ..., y67, z0, z1, z2, z3, ..., z67 | Coordinate points in the world coordinate space in millimeters, with the camera located at (0,0,0) |
|  |  |  |
|  |  |  |
| Facial Action Units (AU) | AU01_r, AU02_r, AU04_r, AU05_r, AU06_r, AU09_r, AU10_r, AU12_r, AU14_r, AU15_r, AU17_r, AU20_r, AU25_r, AU26_r, AU04_c, AU12_c, AU15_c, AU23_c, AU28_c, AU45_c | The suffix "_r" indicates the regression output of the corresponding action unit index; the suffix "_c" represents the binary label of the corresponding action unit index, with 1 indicating its presence and 0 indicating its absence |
|  |  |  |
|  |  |  |
| Eye Gaze Direction Features | x_0, y_0, z_0, x_1, y_1, z_1, x_h0, y_h0, z_h0, x_h1, y_h1, z_h1 | The first two vectors represent the gaze direction of the two eyes in the world coordinate space, while the last two vectors represent the spatial coordinates of the head |
| Head Pose | Tx, Ty, Tz, Rx, Ry, Rz | Tx, Ty, Tz represent the position coordinates, while Rx, Ry, Rz represent the head rotation coordinates in the world coordinate space, measured in millimeters |

**Table S2** Specific input audio features for the SFML model

| Feature Category | Audio Features | Feature Description |
| --- | --- | --- |
| Fundamental Frequency Feature | Mean F0 | Captures the pitch or tone of the sound signal |
|  | Standard deviation of F0 | Indicates the variability in emotional expression |
|  | Minimum F0、Maximum F0 | Identifies the range of pitch in the sound signal |
| Harmonic-to-Noise Ratio Feature | Mean HNR | Detects changes in sound clarity |
|  | Standard deviation of HNR | Measures the stability of the sound signal |
| Spectral Slope Feature | Mean spectral slope | Describes the spectral tilt of the sound signal |
|  | Standard deviation of spectral slope | Detects irregularities in spectral characteristics |
| Mel-Frequency Cepstral Coefficients Feature | Mean of MFCCs | Provides spectral information of the sound signal |
| Acoustic Environment Features | Mean of Signal-to-Noise Ratio | the sound environment the patient may be exposed to |
|  | Standard deviation of Signal-to-Noise Ratio |  |

**Table S3** Fine-tuning the text features used by the EmoBertA model

| Feature Category | Feature Description | Examples |
| --- | --- | --- |
| Emotional Expression | Negative Emotions | “"Sadness," "depression," "despair," "loneliness," "suppression," etc. |
|  | Emotional Intensity | "Pain," "helplessness," "worthlessness," etc |
| Self-Evaluation | Self-Deprecation | "I am a failure," "I am worthless," "I am a complete loser," "I have no value," etc |
|  | Self-Blame | "I have harmed my family, they would be better off without me," "I feel guilty for every mistake, I believe I trouble others," etc |
| Physiological Symptoms | Fatigue and Lack of Energy | "I sleep a lot but still feel tired," "I feel tired and sleepy all the time," etc |
|  | Sleep Problems | "I lie in bed for a long time without falling asleep," "I wake up in the middle of the night and have trouble falling back asleep," "I have shallow sleep and easily wake up with nightmares," etc |
|  | Appetite Changes | "Lack of appetite, no desire to eat," "I don't feel like eating things I used to enjoy," etc |
|  | Digestive Issues | "Stomach pain," "bloating," "constipation," "diarrhea," etc |
|  | Chronic Pain | “"Chronic headaches," "muscle pain," "back pain," etc |
| Social Issues | Feelings of Isolation | "I don't want to see people," "I don't want to socialize with others," etc |
|  | Social Avoidance | "I don't want to interact with friends or family," "I am afraid of social interactions," "I don't want to answer calls or reply to messages," etc |
| Suicidal Tendencies | Suicidal Thoughts | "I feel life is meaningless, I would rather be dead," etc |
|  | Suicidal Plans | "I have taken sleeping pills but didn't succeed," etc |
